# Apolipoprotein C3 facilitates internalization of cationic lipid nanoparticles into bone marrow-derived mouse mast cells

**DOI:** 10.1038/s41598-022-25737-7

**Published:** 2023-01-09

**Authors:** Syed Benazir Alam, Feng Wang, Hui Qian, Marianna Kulka

**Affiliations:** 1 Nanotechnology Research Centre, National Research Council Canada, 11421 Saskatchewan Dr NW, Edmonton, AB T6G 2M9 Canada; 2 Department of Medical Microbiology and Immunology, University of Alberta, Edmonton, Canada

**Keywords:** Biotechnology, Cell biology, Immunology, Nanoscience and technology

## Abstract

Mast cells (MCs), are hematopoetically-derived secretory immune cells that release preformed as well as de novo synthesized inflammatory mediators in response to activation by several stimuli. Based on their role in inflammatory responses, particularly in the lung and skin, MCs provide an effective target for anti-inflammatory therapeutic strategies. Drug-delivery of lipophilic payloads to MCs can be challenging due to their functionally distinct intracellular structures. In the present study, pH-sensitive cationic lipid-based nanoparticles (LNPs) composed of DODMA, DODAP or DOTAP lipids that encapsulated a GFP or eGFP plasmid were constructed using non-turbulent microfluidic mixing. This approach achieved up to 75–92% encapsulation efficiency. Dynamic light scattering revealed a uniformly sized and homogeneous dispersion of LNPs. To promote cellular internalization, LNPs were complexed with apolipoproteins, amphipathic proteins capable of binding lipids and facilitating their transport into cells. Cryo-TEM analysis showed that LNP structure was differentially modified when associated with different types of apolipoproteins. LNP preparations made up of DODMA or DODMA, DODAP and DOTAP lipids were coated with seven apolipoproteins (Apo A1, B, C3, D, E2, E4 and H). Differentiated bone-marrow derived mouse mast cells (BMMCs) were exposed to apolipoprotein-LNP and internalization was measured using flow cytometry. Out of all the apolipoproteins tested, ApoC3 most efficiently facilitated cellular internalization of the LNP into BMMCs as determined by GFP fluorescence using flow cytometry. These effects were confirmed in a less differentiated but also interleukin-3-dependent model of mouse mast cells, MC/9. ApoC3-LNP enhanced internalization by BMMC in a concentration-dependent manner and this was significantly increased when BMMC were pre-treated with inhibitors of actin polymerization, suggesting a dependence on intracellular shuttling. Activation of peroxisome proliferator-activated receptor gamma (PPARγ) decreased ApoC3-LNP internalization and reduced the expression of apolipoprotein E receptor 2 (ApoER2), suggesting that ApoC3-LNP binding to ApoER2 may be responsible for its enhanced internalization. Furthermore, ApoC3 fails to facilitate internalization of LNPs in *Lrp8*^*−/−*^ KO BMMC that do not express ApoER2 on their cell surface. Altogether, our studies reveal an important role of ApoC3 in facilitating internalization of cationic LNPs into MCs.

## Introduction

Clinical approval of lipid nano particles (LNP) for delivery of nucleic acids and small molecule drugs heralded the advent of nanotechnology-based pharmacotherapy. In particular, this approach has recently been utilized to deliver antigenic payloads to immune cells and initiate protective immune responses in vaccine development. However, LNP delivery to immune cells remains a major unmet challenge, particularly to cell types that are difficult to isolate and culture in vitro. Cationic lipids have several advantages to polymer-based nanoparticles. First, cationic lipids are much more likely to penetrate immune cell membranes because they can interact with the anionic phospholipids in the plasma membrane and thereby promote its uptake via endocytic pathways^1–3^. Second, they are immunologically inert and therefore unlikely to activate nonspecific immune responses^4^. Third, cationic lipids can be easily functionalized to specifically target cell membrane receptors and biomarkers. Although cationic lipid nanoparticles are sometimes synthesized using the double emulsion-solvent evaporation method, it can result in heterogeneous populations of LNPs with differing sizes, compositions and incomplete encapsulation of payloads. We have previously used controlled microfluidic mixing to generate discrete populations of 50–150 nm LNPs with higher encapsulation efficiencies^5^. We have optimized several different formulations based on six specific lipids: 1,2-dioleyloxy-3-dimethylaminopropane (DODMA), 1,2-dioleoyloxy-3-dimethylaminopropane (DODAP), 1,2-dioleoyl-3-trimethylammonium-propane (DOTAP), 1,2-dioleoyl-*sn*-glycero-3-phosphoethanolamine (DOPE), 1,2-dimyristoyl-*sn*-glycero-3-phosphoethanolamine-N-[methoxy(polyethylene glycol)-2000] (PEG-DMPE) and cholesterol. In combination these lipids created stable and effective LNP delivery vehicles.

Although cationic lipids are effective in delivering cargo to adherent cells, they are particularly troublesome for cells grown in suspension due to reduced probability of interaction with the delivery vehicle^6^. Non-transformed, primary cultured immune cells are extremely difficult to transfect due to their low division rate, intrinsic self-defence mechanisms to prevent introduction of foreign genetic material, and increased susceptibility to apoptosis when exposed to cationic lipids^7,8^. These challenges can be overcome by coating LNPs with apolipoproteins (Apo) which bind both lipids and lipoproteins to form polar structures that act as lipid transport mechanisms in the lymph, blood and cerebrospinal fluid^9–12^. Apos can act as intrinsic docking and plasma membrane entry facilitators and are exploited by hepatitis C to enable virus entry^13–16^. Apos have recently been used to create thermostable lipid-like nanoparticles for delivery of RNAi^17^, miRNA^18^, siRNA^19^, and small molecule therapies^20^. These studies demonstrate that targeting of specific tissues appears to be dependent upon the type of Apo or combination of Apos that were used to coat the nanoparticles. It is yet to be demonstrated whether this strategy is effective for the transfection of immune cells, specifically primary cultured immune cells.

Mast cells are a tissue-resident innate immune cell that mediate tissue homeostasis but also facilitates inflammatory disease^21^. Unlike lymphocytes, mast cells are large, granular and extremely difficult to transfect using conventional non-viral strategies^22,23^. We hypothesized that Apos may facilitate the internalization of cationic LNPs into terminally differentiated mouse bone marrow-derived mast cells (BMMC). We have screened seven different Apos (Apo A1, B, C3, D, E2, E4 and H) and showed that ApoC3 significantly enhances the internalization efficiency of two distinct LNP preparations (made up of DODMA or DOTAP, DODAB and DODMA) into BMMC. Internalization efficiency was significantly enhanced when BMMC were pre-treated with actin polymerization inhibitors as well as a significant decrease was observed when PPARγ was activated using agonist GW1929 and troglitazone.

## Materials and methods

### Lipids and chemicals

The lipid 1,2-dioleyloxy-3-dimethylaminopropane (DODMA) was purchased from Cayman Chemical (Ann Arbor, MI, USA), 1,2-dioleoyloxy-3-dimethylaminopropane (DODAP), 1,2-dioleoyl-3-trimethylammonium-propane (DOTAP), 1,2-dioleoyl-*sn*-glycero-3-phosphoethanolamine (DOPE), and 1,2-dimyristoyl-*sn*-glycero-3-phosphoethanolamine-N-[methoxy(polyethylene glycol)-2000] (PEG-DMPE) and cholesterol were obtained from Sigma–Aldrich Canada (Oakville, ON, Canada). Anhydrous ethanol was obtained from Commercial Alcohols (Brampton, ON, Canada).

### Plasmid DNA

The plasmid pMIPS-VEE V 34 expressing a GFP gene (6.3 Kb) was a gift from Defence Research and Development Canada. The plasmid peGFP which expresses enhanced green fluorescent protein (eGFP) (4.7 Kb) from a human cytomegalovirus immediate early promoter was purchased from Clontech BD Biosciences (Mississauga, ON, Canada)^5^. All plasmids were isolated using GeneJET Endo-free plasmid Maxiprep or PureLink Expi Endotoxin-free Maxi Plasmid Purification Kits (ThermoFisher Scientific, Burlington, ON, Canada) as per the manufacturer’s instructions.

### Generation of LNPs

All LNPs were prepared by combining appropriate volumes of cationic lipid (DODMA alone (DODMA-LNPs)or a combination of DODMA, DODAP and DOTAP lipids (Hydrid LNPs); 50 mol% unless otherwise stated), helper lipid (DOPE; 11.5 mol%), PEGylated lipid (PEG-DMPE; 1.0 mol%; unless otherwise stated), and cholesterol (37.5 mol%; unless otherwise stated) in anhydrous ethanol with plasmid DNA in 25 mM sodium acetate (pH 4.0) at a nitrogen to phosphorus (N/P) ratio of 6:1 using the NanoAssemblr Benchtop instrument and microfluidic cartridges (Precision Nanosystems, Vancouver, BC, Canada) set to a combined flow rate of 9 ml/min [flow rate ratio of 3 (DNA/aqueous) to 1 (ethanol/lipid)]. The total lipid concentration was maintained at 14.68 mM and the cholesterol content was varied to offset changes in cationic lipid or PEGylated lipid content. The LNP formulations were diluted with PBS (pH 7.4, without magnesium and calcium; Gibco) and subjected to three sequential diafiltration steps using Amicon Ultra centrifugal filters with a nominal molecular weight limit of 10 kDa (Millipore, Etobicoke, ON, Canada)^5^.

### Characterization of LNPs

The Z-average of each LNP formulation in PBS was determined using dynamic light scattering (DLS) with a Zetasizer Nano ZS (Malvern Instruments, Malvern, Worcs, UK) set to 25 °C and a measurement angle of 173°. Samples were diluted 20 times with PBS for instrument optimization.

DNA encapsulation efficiencies were measured using a Quant-iT PicoGreen dsDNA Assay Kit (ThermoFisher Scientific)^5^. Briefly, the amount of PicoGreen fluorescence associated with free (unencapsulated) DNA (Ff) in the LNP solution was compared to the level of PicoGreen fluorescence obtained following a 5 min lysis of the LNPs using 0.05% Triton X-100 (total fluorescence, Ft). The unlysed and lysed LNP solutions (100 µl) were combined with 100 µl 1:200 PicoGreen solution prior to measurement (final [Triton X-100] = 0.025%). Standard curves containing pMIPS-VEE V 34 or eGFP plasmid DNA in the presence or absence of 0.025% Triton X-100 were also generated. Fluorescence was measured using a Synergy H1M plate reader (BioTek, Winooski, VT, USA) and the percentage of DNA encapsulation was calculated with the following formula: (Ft – Ff)/Ft × 100.

The morphology of LNPs in each formulation was characterized in JEOL2200FS TEM with an in-column Omega energy filter, running at 200 kV accelerating voltage. The cryo-TEM specimen of LNPs in aqueous solution were prepared using plunge freezing method. Briefly, 4 µl of LNPs solution was deposited on lacey carbon film supported TEM grids, glow discharged prior to placing the solution on and excess solution was blotted away from TEM grids using a filter paper. TEM grids were rapidly plunged into liquid ethane at − 180 °C (Leica EM GP2 Plunge Freezer) and transferred to a storage box in liquid nitrogen until imaging. Bright field cryo-TEM images of LNPs embedded in vitreous ice were obtained at − 180 °C. A 10-eV energy slit and defocusing were applied to enhance the contrast of LNPs and a low dose imaging was used to mitigate electron beam damage.

### Cell culture

#### BMMC

All animals were sacrificed in accordance with the Canadian Council on Animal Care Guidelines and Policies (https://ccac.ca/en/about-the-ccac/) with approval from the Health Science Animal Care and Use Committee for the University of Alberta and the University of Cincinnati College of Medicine. The study is reported in accordance with ARRIVE guidelines (https://arriveguidelines.org). Femurs were removed from 12-week-old C57Bl/6 mice, or C57Bl/6; *Lrp8*^*−/−*^ mice^24^ (a kind gift from Dr. David Y. Hui, University of Cincinnati College of Medicine) using standard dissection. Bone marrow was aspirated using a 27 gauge needle and the cells were cultured in RPMI media (Fisher, Hampton, New Hampshire, USA) supplemented with 4 mM l-glutamine (Fisher), 50 μM BME (Sigma-Aldrich, Oakville, Ontario, Canada), 1 mM sodium pyruvate (Fisher), 100 U/ml penicillin/100 μg/ml streptomycin (Fisher), 0.1 mM nonessential amino acids (Fisher), 25 mM HEPES (Fisher), 10% FBS (Gibco, Burlington, Ontario, Canada) and 30 ng/ml mouse recombinant interleukin (IL)-3 (Peprotech, Rocky Hill, New Jersey, USA), pH 7.4–7.6, in a humidified atmosphere of 5% CO_2_ in air at 37 °C. This media will be referred to as “supplemented RPMI”. The cell suspensions were maintained at a density of 0.25–0.5 × 10^6^ cells/ml for 4 weeks when the cells were tested for FcεRI and c-Kit expression by flow cytometry to confirm maturation. After 4 weeks, 99% of cells were double positive for c-Kit and FcεRI (Fig. 2A-ii). BMMC were used between 4 and 8 weeks of age.

#### MC/9

MC/9 cells were cultured in supplemented RPMI and maintained at a density of 0.25–0.5 × 10^6^ cells/ml. The cells were used up to 15 weeks of culture from time of removal from cryopreservation.

### Flow cytometric analysis of FcεRI and Kit expression and GFP fluorescence

BMMC or MC/9 were suspended in phosphate-buffered saline (PBS) supplemented with 0.5% bovine serum albumin (BSA) (PBS-BSA, Calbiochem Omnipur BSA fraction V) at 1.5 × 10^6^ cells/ml, and incubated with 0.006 µg/ml CD117 (c-Kit) PE (eBioscience, San Diego, California, USA) and 0.006 μg/ml FcεRIα APC (eBioscience) for 1 h at 4 °C. After washing with PBS-BSA twice, cells were re-suspended in 100 μl 0.5% BSA/0.05% sodium azide in PBS (PBS-BSA-sodium azide) and analyzed on a CytoFlex flow cytometer (Bechman Coulter, USA) by acquiring 20,000 events. Rat IgG2b κ PE (eBioscience) and Armenian Hamster IgG APC (eBioscience) were used as isotype controls.

To measure GFP fluorescence, BMMC (WT or *Lrp8*^*−/−*^ KO) or MC/9 were washed twice with PBS-BSA and resuspended in 100 µl PBS-BSA-sodium azide and analyzed on a Cytoflex flow cytometer (Beckman Coulter, USA) equipped with an Argon ion laser (488–514 nm) and bandpass filter to enable detection fluorescence emission at 516 nm (for GFP), 578 nm (for c-Kit PE) and 660 nm (for FcεRIα APC). 20,000 events per sample were acquired at a flow rate of 30 µl/min at room temperature. As mast cells are very granular (high SSC) and large (high FSC) when compared to other immune cell types such as monocytes and lymphocytes, a well-defined cell population containing relatively high FSC and SSC were gated and analyzed as shown in Fig. 2A-i (BMMC) and 2F-i (MC/9). Data was generated using FlowJo 10.6.2 software (Becton, Dickinson and Company, USA).

### LNP and apolipoprotein complex formation

LNPs and Apos were incubated in ddH_2_O water overnight at 4 °C. LNPs equivalent of 5 µg encapsulated plasmid DNA were incubated with 3 µg of each of the different human Apos; ApoA1 (Sigma, SRP4693), ApoB (Sigma, 178456), ApoC3 (Sigma, A3106), ApoD (Sigma, SRP4326), ApoE2 (Peprotech, 350-12), ApoE4 (Peprotech, 350-04) and ApoH (Sigma, G9173), unless otherwise stated, in a final volume of 200 µl. For control experiments (i.e. LNP only), LNPs were incubated with ddH_2_O water (in the absence of any apolipoprotein) overnight at 4 °C in a final volume of 200 µl. For Cryo TEM reactions were set up as described above in the absence of ddH_2_O.

### Transfection

BMMC were treated with Apo-LNP mixture (200 µl) in serum free supplemented RPMI media in a total volume of 500 µl. After four hr, 500 µl of supplemented RPMI media was added to the cell suspension, so that the final concentration was 0.1 × 10^6^ cells/ml, 5 µg plasmid/ml and 3 µg Apo/ml. Control cells (BMMC or MC/9) were treated with 200 µl ddH_2_O water. The plate was incubated in a humidified 5% CO_2_ incubator at 37 °C for 24 h followed by cell collection and processing for flow cytometry as described above.

### Endocytosis inhibitor and PPARγ experiments

BMMC (1 × 10^6^ cells/ml) were treated with cytochalasin B (0.3 µM), 3-methyladenine (600 µM), chloroquine (20 nM), latrunculin B (3 µM) or a cocktail consisting of all the inhibitors for 1 h at 37 °C prior to treatment with ApoC3-LNP mixture. Samples were collected at 24 h post transfection and were processed for flow cytometry as described above.

BMMC (1 × 10^6^ cells/ml) were treated with the PPARγ agonist GW1929 (10 µM) or troglitazone (5 µM), PPARγ antagonist GW9662 (5 µM) or DMSO for 1 h at 37 °C prior to treatment with the ApoC3-LNPs. Samples were collected at 24 h post transfection and were processed for flow cytometry as described above.

### Trypan blue staining

0.1 × 10^6^ BMMC were incubated with 5 µg plasmid equivalent of LNP’s for 24 h at 37 °C followed by trypan blue staining to measure % viability. Cell numbers were counted on a hemocytometer and analyzed under a light microscope (Leica MiroSystems CMS GmbH, Ernst-Leitz-ste, Model DMi1, China).

### β-Hexosaminidase assay

BMMC (100,000 cells/treatment) were suspended in HEPES buffer (10 mM HEPES, 137 mM NaCl, 2.7 mM KCl, 0.4 mM Na_2_HPO_4_·7H_2_O, 5.6 mM glucose, 1.8 mM CaCl_2_·2H_2_O, 1.3 mM MgSO_4_·7H_2_O, pH 7.4) and treated with 1, 5 or 10 µg/ml ApoC3 for 45.

minutes. Degranulation was determined by measuring the amount of β-hexosaminidase released into the supernatants and normalized to the amount of β-hexosaminidase remaining in the cells after the 45 min incubation period. β-hexosaminidase was quantified by measuring the hydrolysis of *p*-nitrophenyl N-acetyl-β-d-glucosamide (Sigma-Aldrich) in 0.1 M sodium citrate buffer (40 mM citrate, 20 mM Na_2_HPO4·7H_2_O, 20 mM Na_2_HPO_4_, pH 4.5) for 60 min at 37 °C. The percentage of β-hexosaminidase release was calculated as a percent of total content. BMMC activated with the calcium ionophore, A23187 (1 µM) was included as a positive control.

### ELISA analysis of TNF production

WT BMMC or *Lrp8*^*−/−*^ KO BMMC (1 million cells/ml) were treated with ApoC3, ApoB, ApoE2, and ApoE4 (all at 10 µg/ml) or A23187 (1 µM) for 24 h at 37 °C and TNF production was measured in cell-free supernatants using a commercial ELISA (R&D Systems, Minneapolis, MN, USA).

### RNA extraction and cDNA synthesis

BMMC or MC/9 were treated with the PPARγ agonist GW1929 (10 µM) or troglitazone (5 µM), and antagonist GW9662 (5 µM) or DMSO for 24 h at 37 °C followed by RNA extraction using the Qiagen RNeasy Mini Kit (Cat# 74106) that employed the on-column DNase (Qiagen RNase-Free DNase Set, Cat# 79254) digestion.

The purity and concentration of the RNA was determined using the Nanodrop One (Thermo Scientific) and cDNA was synthesized using 1 µg of total RNA utilizing the High capacity cDNA reverse transcription kit (4368814, Applied Biosystems).

### qPCR

qPCR was performed utilizing 20 ng of cDNA, Fast SYBR Green master mix (438612, Applied Biosystems), gene specific IDT oligonucleotide primers as described in Table 1 and a StepOnePlus real time PCR machine (Applied Biosystems).

**Table 1:** Intron spanning mouse oligonucleotide primers used in this study.

Gene ID	Gene name	Abbreviation	Forward primer	Reverse primer
NM_001080926	Low density lipoprotein receptor-related protein 8	Lrp8	CATCGTGCCCATAGTGGTAATA	GCTCATCTTCCTCTTCCTCTTC
NM_008084.3	Glyceraldegyde-3-phosphate dehydrogenase	Gapdh	GGGTTCCTATAAATACGGACTGC	CCATTTTGTCTACGGGACGA

### Protein extraction and quantification

BMMC (1 × 10^6^ cells) were collected by centrifugation at 500×*g* for 10 min and the cell pellet was washed once in cold PBS. BMMC were lysed with 100 µl of 1 × RIPA lysis and extraction buffer (PI89900, Thermo Scientific™) containing protease and phosphatase inhibitor cocktail (PPC1010, Sigma-Aldrich). The lysate was gently rocked on a shaker for 60 min at 4 °C followed by centrifugation at ∼14,000×*g* for 15 min. The supernatant was utilized for further analysis. The protein concentration was determined by BCA assay (PI23225, Thermo Scientific™, Pierce™ BCA Protein Assay Kit).

### Western blot analysis

Twenty micrograms of protein from each sample was electrophoresed on Bolt™ 4 to 12%, Bis-Tri Protein Gel (NW04122BOX, Thermo Scientific™, Invitrogen™) and electroblotted onto a nitrocellulose membrane (LC2000, Thermo Scientific™, Invitrogen™). The membrane was blocked with intercept (TBS) blocking buffer (LiCOR Catalog No. 927-60001) for 1 h at room temperature (RT), and then incubated for 3 h at RT with antibodies against ApoER2 (ab108208, Abcam for detecting ApoER2 in MC/9 lysates; A03444-2, Boster Bio for detecting ApoER2 in BMMC lysates) and β-Actin (5441, Sigma-Aldrich). The membrane was washed three times with TBS buffer and then incubated with IRDye800CW goat anti-rabbit (827-08-365, LiCOR) and IRDye680RD goat anti-mouse (926-68170, LiCOR) secondary antibodies for 1 h at room temperature. The membrane was washed three times with TBS buffer and fluorescence was detected using the LiCOR Odyssey CLX Imaging System. The samples presented in Fig. 5 were run on the same gel, blotted on the same membrane, using the same amount of total protein to allow for comparisons between the two cell types. 

### Fluorescence microscopy

WT BMMC or *Lrp8*^*−/−*^ KO BMMC were fixed with 4% paraformaldehyde for 10 min, permeabilized with 0.1% Triton X-100 for 5 min, and blocked with 1% BSA-PBS for 30 min. Cells were then incubated with anti-ApoER2 polyclonal antibody (ab108208, Abcam) at RT for 1 h and followed by co-incubation with the secondary antibody (Alexa Fluro 594, anti-rabbit IgG) and DAPI dye solution (1:1000 dilution of a 1 mg/ml solution, 62248, ThermoFisher Scientific) for 45 min at RT in the dark. Images were acquired using the Echo Revolve 4 Hybrid Fluorescence microscope (San Diego, CA, USA).

### Statistical analysis

Experiments were conducted at least in triplicate using three independent BMMC or MC/9 cultures started from three animals and values represent mean of n = 3, 4 or 5 ± standard error of the mean. P values were determined using Student t test and data was analyzed using GraphPad Prism software (https://www.graphpad.com/quickcalcs/ttest1.cfm).

### Ethics approval and consent to participate

All animals were sacrificed in accordance with the Canadian Council on Animal Care Guidelines and Policies (https://ccac.ca/en/about-the-ccac/) with approval from the Health Science Animal Care and Use Committee for the University of Alberta and the University of Cincinnati College of Medicine.

## Results

### Physicochemical properties of LNP formulations

Cationic lipid-based nanoparticles (LNP) composed of DODMA lipids and a 6.3 Kb (GFP-DODMA-LNP) or 4.7 kb (eGFP-DODMA-LNP) plasmid DNA expressing GFP and enhanced GFP respectively, were synthesized using microfluidic mixing. Another LNP formulation (GFP-Hybrid-LNP) was generated from three different cationic lipids: DODMA, DODAP, DOTAP and encapsulating a 6.3 Kb plasmid DNA expressing GFP (Table 2). The LNP formulations were generated by combining a 1:3 molar ratio of the lipids with the aqueous phase. PicoGreen analysis showed a robust > 71% encapsulation efficiency of the DNA payload for all the formulations (Table 2). Dynamic light scattering (DLS) analysis indicated that the majority of the LNPs in various formulations (such as GFP-DODMA-LNP, eGFP-DODMA-LNP or GFP-Hybrid-LNP) had a Z-average (average diameter) of approximately > 127 nm and < 190 nm. Moreover, the LNPs generated by microfluidic mixing were homogeneous with a polydispersity index (PDI) measuring close to 0.1. The overall negative or positive zeta potential of the LNP preparations suggest that the particles had an overall net negative or net positive charges respectively on their surface, indicative of net repulsive force between individual LNPs in solution to attain physical stability (Table 2).

**Table 2:** Physicochemical properties of LNPs and Apo-LNP complex.

Formulation name and abbreviation	Composition	Ratio of organic to aqueous phase	Z average (nm)	Zeta potential (mV)	PDI	Encapsulation efficiency (%)
GFP-DODMA-LNP *(GFP-LNP)*	DODMA, DOPE, Cholesterol, PEG2000-PE	1:3	127.2–189.3	(−) 4.68	0.105	71.35–97.25
No DNA-DODMA-LNP control	DODMA, DOPE, Cholesterol, PEG2000-PE	1:3	154.6	(−-) 3.17	0.139	NA
GFP-Hybrid-LNP *(GFP-Hy-LNP)*	DODMA/DODAP/DOTAP, DOPE, Cholesterol, PEG2000-PE	1:3	161	(−) 7.11	0.087	92
No DNA-Hybrid-LNP control	DODMA/DODAP/DOTAP, DOPE, Cholesterol, PEG2000-PE	1:3	140	(−) 1.44	0.139	NA
eGFP-DODMA-LNP *(eGFP-LNP)*	DODMA, DOPE, Cholesterol, PEG2000-PE	1:3	136–180.73	(−) 1.67–(+) 18.80	0.107–0.206	88.57–94.5
ApoC3-eGFP-DODMA-LNP	ApoC3 + eGFP-DODMA-LNP	NA	137.9	(−) 7.52	0.103	NA
ApoB-eGFP-DODMA-LNP	ApoB + eGFP-DODMA-LNP	NA	287.53	(+) 8.95	0.187	NA
ApoE2-eGFP-DODMA-LNP	ApoE2 + eGFP-DODMA-LNP	NA	186.97	( +) 18.33	0.186	NA
ApoE4-eGFP-DODMA-LNP	ApoE4 + eGFP-DODMA-LNP	NA	207.90	(+) 15.90	0.212	NA

### Characterization of LNP and Apo-LNP complex formulations using DLS and picogreen analysis

Physicochemical properties of LNPs were determined as described in the “Materials and methods”. For Apo-LNP complexes, 5 µg plasmid equivalent LNPs were incubated with 3 µg ApoC3, ApoB, ApoE2 and ApoE4 overnight at 4 °C followed by DLS analysis.

**Figure 1:**
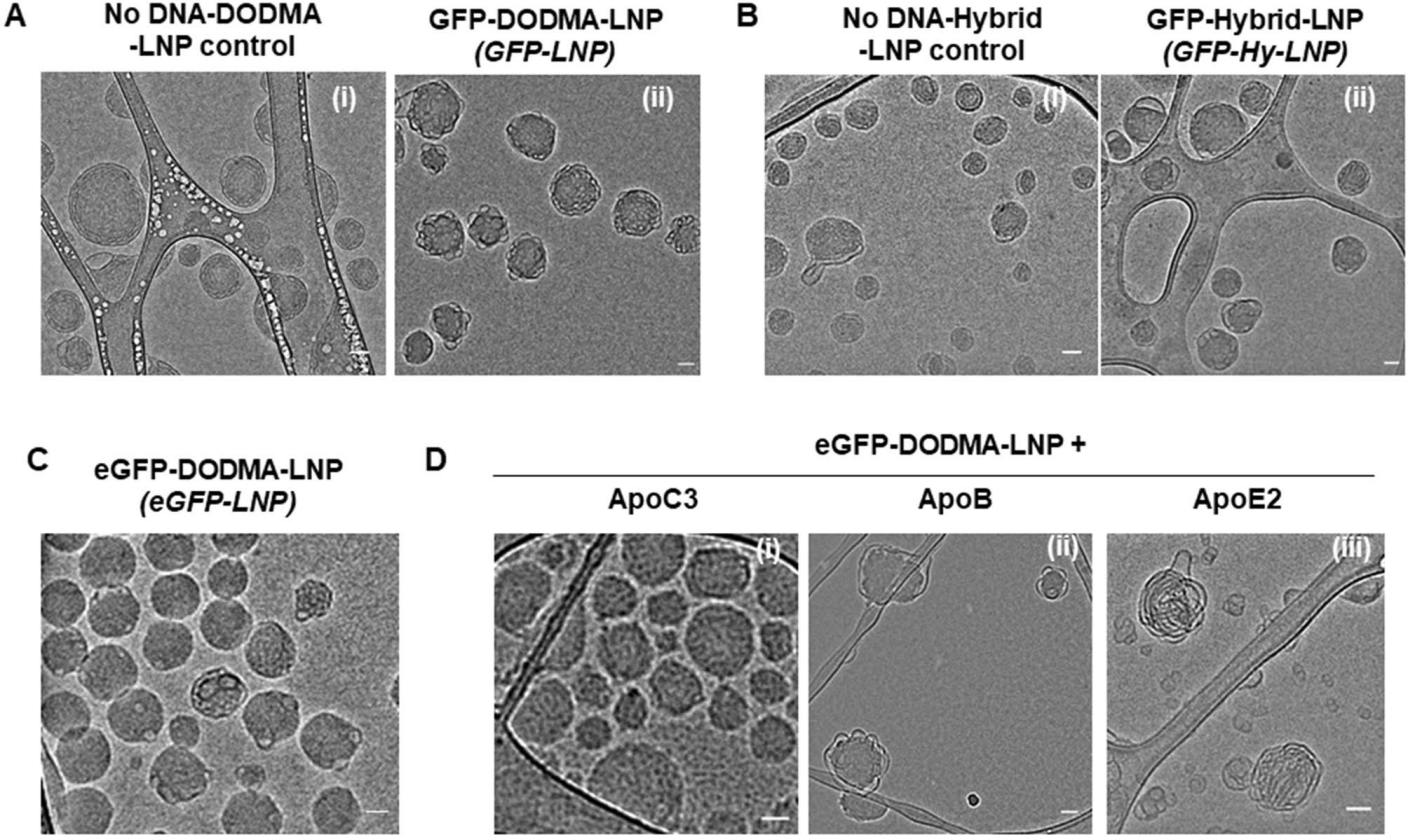
Cryo-TEM analysis of LNPs. (**A**) Cryo TEM analysis of No DNA-DODMA-LNP control (**A-i**) or GFP-DODMA-LNP (**A-ii**), No DNA-Hybrid-LNP control (**B-i**) or GFP-Hybrid-LNP (**B-ii**) and eGFP-DODMA-LNP (**C**). 5 µg plasmid equivalent eGFP-DODMA-LNPs were incubated with 3 µg ApoC3 (**D-i**), ApoB (**D-ii**) and ApoE2 (**D-iii**) overnight at 4 °C followed by Cryo-TEM analysis. Scale bar represents 50 nm.

Cryo TEM analysis (Fig. 1A,B) showed that LNPs in all the formulations are “sponge” like mesophase structured particles rather than solid particles. DODMA-LNPs with smaller plasmid DNA (eGFP-DODMA-LNPs with 4.7 kb plasmid DNA, Fig. 1C) are denser than those with larger plasmid DNA (GFP-DODMA-LNPs with 6.3 kb plasmid DNA, Fig. 1B). Also, LNPs without plasmid DNA have more electron dense core compared to LNPs encapsulating plasmid DNA (Compare images in Fig. 1A-i and ii or B-i and ii).

To evaluate the changes in the structure and physicochemical properties of LNPs in the presence of Apos known to bind lipids, eGFP-DODMA-LNPs were complexed with ApoC3 (since it facilitates LNP internalization, see below). Cryo-TEM analysis showed that the ApoC3 (Fig. 1D-i) were entrapped on the lipid surface or mesoporous matrix of the eGFP-DODMA-LNPs, making them appear slightly larger compared to eGFP-DODMA-LNPs alone. To evaluate if this feature was specific to ApoC3, eGFP-DODMA-LNPs were complexed with ApoB and ApoE2 followed by cryo-TEM analysis. Each of the Apo-LNP complexes displayed unique structural characteristics (Fig. 1D). Whereas the ApoC3-LNP appear dense with a defined outer edge and electron dense inner core, ApoB-LNP appears to have a less organized outer surface with projections and a less dense core. The ApoE2-LNP were the least organized, similar to balls of a long string-like structure and no definitive core.

### Apolipoprotein C3 facilitates internalization of GFP-DODMA-LNP into BMMC.

**Figure 2:**
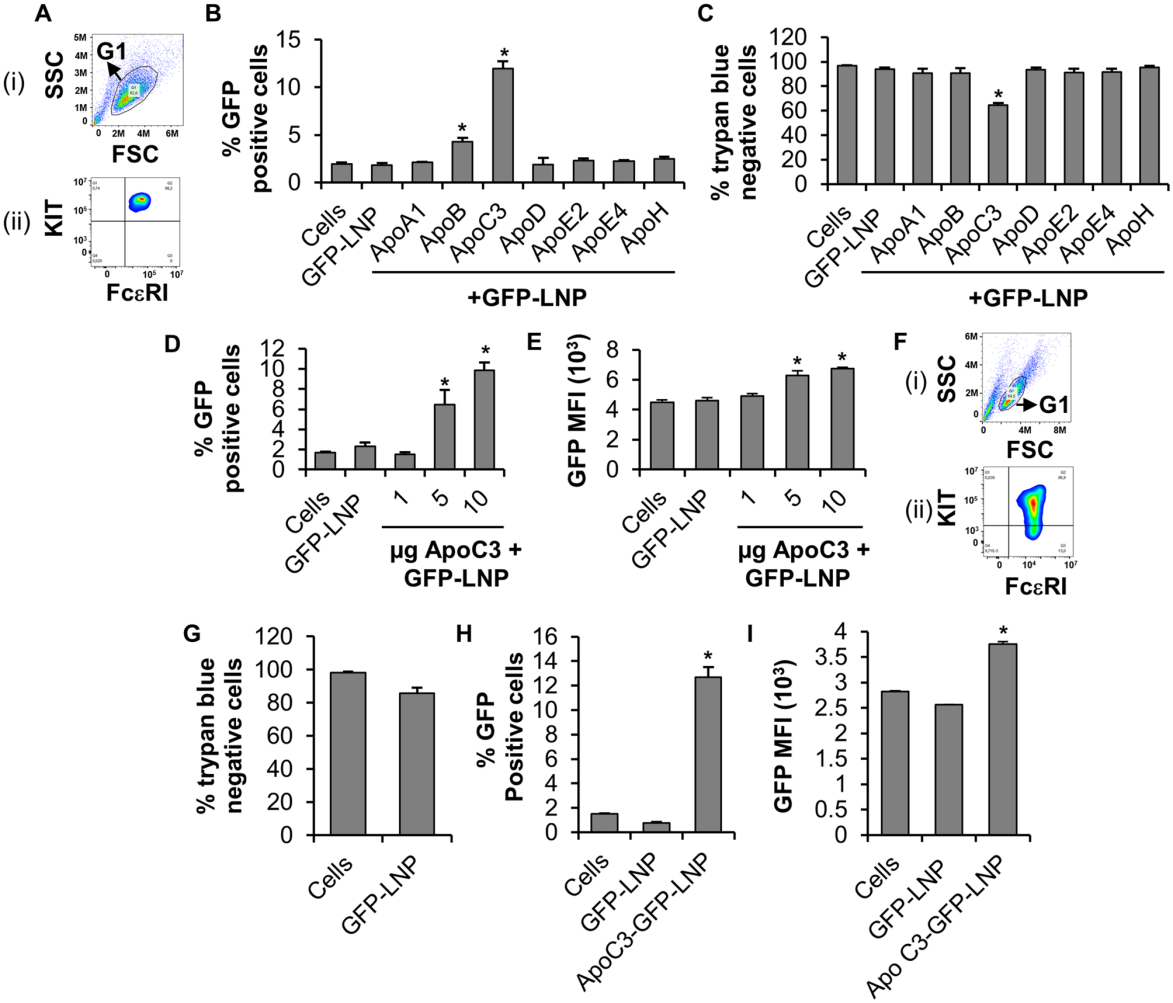
ApoC3 facilitates internalization of GFP-DODMA-LNP (GFP-LNPs) into BMMC and MC/9. (**A**) Gating strategy used to exclude debris/dead cells from flow cytometric analysis of terminally differentiated BMMC co-expressing FcεRI and KIT receptors. (**B**, **C**) BMMC were treated with 3 µg Apo (ApoA1, B, C3, D, E2, E4 and H)-GFP-LNP’s or GFP-LNP alone followed by flow cytometry (**B**) or trypan blue staining (**C**). Cells represent control BMMC that have not been treated with GFP-LNP or Apo-GFP-LNP. (**D**, **E**) BMMC were transfected for 24 h with ApoC3 (1–10 µg)-GFP-LNP’s followed by flow cytometry to determine % GFP positive cells (**D**) and GFP mean fluorescence intensity (MFI) (**E**). (**F**) Gating strategy used to exclude debris/dead cells from flow cytometric analysis of MC/9 co-expressing FcεRI and KIT receptors. (**G**) MC/9 were treated with GFP-LNP for 24 h followed by trypan blue staining. Cells represent control MC/9 that have not been treated with GFP-LNP. (**H**, **I**) MC/9 were transfected with 3 µg ApoC3-GFP-LNP followed by flow cytometry to determine % GFP positive cells (**H**) and GFP MFI (**I**). Cells represent control MC/9 that have not been treated with GFP-LNP or ApoC3-GFP-LNP. n = 3. p < 0.01 (*) is relative to GFP-LNP.

Since, Apos are known to bind lipids for facilitating their transport and metabolism in human circulation^9,25^, we tested seven different Apos (Apo A1, B, C3, D, E2, E4 and H) for their ability to facilitate internalization of GFP-DODMA-LNP into BMMC. Highly pure populations of terminally-differentiated BMMC expressing both CD117 (KIT) and FcεRI (Fig. 2A-ii) were treated with GFP-DODMA-LNP or Apo-GFP-DODMA-LNPs for 24 h and the GFP expression was measured by flow cytometry. Transfection efficiency was measured as % GFP positive cells. GFP-DODMA-LNPs were not toxic to BMMCs as determined by trypan blue exclusion assay (Fig. 2C, compare % viability of Cells and LNP). ApoB and ApoC3 significantly enhanced internalization of GFP-DODMA-LNPs into BMMC (Fig. 2B, p < 0.05) and ApoC3 gave the best relative transfection efficiency out of all the tested Apos with 12 ± 0.7% GFP positive cells compared to cells that have been treated with just the GFP-DODMA-LNPs having a background fluorescence of 1.8%. ApoC3 decreased BMMC viability by 30% (Fig. 2C, compare % trypan blue negative cells between LNP and ApoC3 + LNP), thus all the flow cytometric analysis only considered cells having a high side scatter (SSC) and forward scatter (FSC) value (gated cells, G1; Fig. 2A-i) which represented the healthy, unaltered cell population.

### Apolipoprotein C3 facilitates internalization of GFP-DODMA-LNPs in a concentration-dependent manner.

Since, ApoC3 was the most efficient in facilitating internalization of GFP-DODMA-LNP cargo into BMMC, we determined whether increasing concentrations of ApoC3 would lead to enhanced transgene delivery and expression. When increasing concentrations of ApoC3 (1, 5 and 10 µg) were incubated with GFP-DODMA-LNP, followed by transfection into BMMC, we found that there was a significant increase in % GFP positive cells with 5 and 10 µg of ApoC3 (Fig. 2D, p < 0.05; Supplementary Fig. 1A) and a concentration-dependent increase in GFP mean fluorescence intensity (MFI) (Fig. 2E).

### Apolipoprotein C3 facilitates internalization of GFP-DODMA-LNP into MC/9.

Since BMMC are considered to be an end-stage terminally differentiated myeloid-derived immune cells, we tested the internalization efficiency of GFP-DODMA-LNPs into a rapidly-dividing transformed mouse mast cell line called MC/9. MC/9 are similar to BMMC in that they express FcεRI (Fig. 2F-ii). When MC/9 cells were incubated with GFP-DODMA-LNPs for 24 h, a 14% reduction in cell viability was observed (Fig. 2G). MC/9 cells were treated with GFP-DODMA-LNP or ApoC3-GFP-DODMA-LNPs for 24 h and the GFP expression was measured by flow cytometry. For analysis purpose, healthy and unaltered cell population having high FSC and SSC (gated cells, G1; Fig. 2F-i) were considered to preclude the fluorescence emitted by dead cells/debris. ApoC3 significantly enhanced transfection efficiency of GFP-DODMA-LNPs into MC/9 to about 12.6 ± 0.8% when compared to cells treated with GFP-DODMA-LNP alone as determined by the % GFP positive cells (Fig. 2H), GFP MFI (Fig. 2I) and dot plot (Supplementary Fig. 1B) analysis.

### Apolipoprotein C3 facilitates internalization of GFP-Hybrid-LNP (composed of DODMA, DOTAP and DODAP lipids) into BMMC

**Figure 3:**
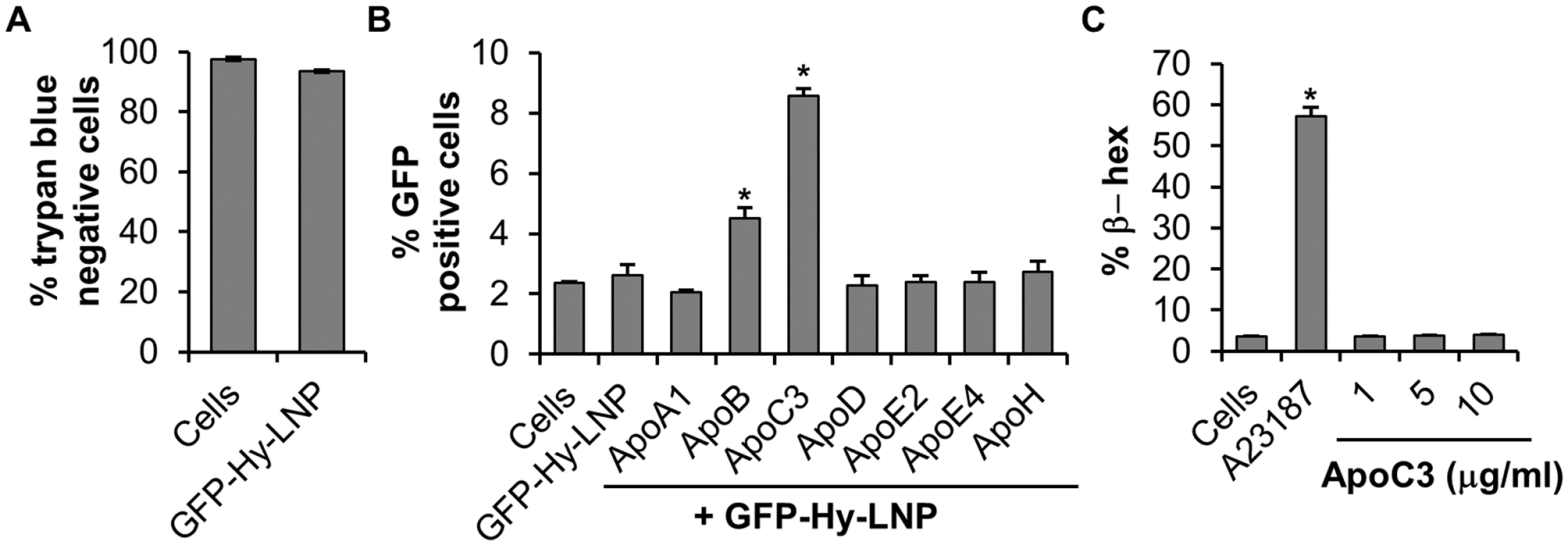
ApoC3 facilitates internalization of GFP-Hybrid-LNP (GFP-Hy-LNP) into BMMC. (**A**) BMMC were treated with GFP-Hy-LNP for 24 h then stained with trypan blue. Cells represent control BMMC that have not been treated with GFP-Hy-LNP. (**B**) BMMC were transfected with 3 µg Apo (ApoA1, B, C3, D, E2, E4 and H)—GFP-Hy-LNP’s or GFP-Hy-LNP alone followed by flow cytometry to determine % GFP positive cells. Cells represent control BMMC that have not been treated with GFP-Hy-LNP or Apo-GFP-Hy-LNP. (**C**) BMMC were treated with 1, 5 or 10 μg/ml ApoC3 for 45 min followed measuring degranulation by calculating the % release of β-hexosaminidase (β-hex). A23187 activated BMMC was include as a positive control. Cells represent control BMMC that have not been treated with A23187 or ApoC3 (n = 4). p < 0.01 (*) is relative to GFP-Hy-LNP (**B**) or cells (**C**) respectively.

Next, we tested whether ApoC3 promoted internalization efficiency of GFP-Hybrid-LNP cargo composed of three different cationic lipids (DODMA, DOTAP and DODAB). Trypan blue exclusion assay showed that the GFP-Hybrid-LNP’s reduced BMMC viability by only 4% (Fig. 3A).

BMMC were transfected with GFP-Hybrid-LNPs or Apo-GFP-Hybrid-LNPs for 24 h and the GFP expression was measured by flow cytometry. Consistent with the observation that ApoB and ApoC3 facilitated the internalization of GFP-DODMA-LNPs, ApoB (4.5%) and ApoC3 (8.6%) significantly increased internalization of GFP-Hybrid-LNP into BMMC as determined by an increase in the % GFP positive cells (Fig. 3B, p < 0.05) when compared to cells treated with just the GFP-Hybrid-LNP (2.6%).

Since the overall transfection efficiency of GFP-DODMA-LNPs (Fig. 2B, 10.2%) was greater than that observed with the GFP-Hybrid-LNPs (Fig. 3B, 5.96%), subsequent experiments were performed with GFP-DODMA-LNPs.

### Apolipoprotein C3 does not activate BMMC degranulation

The results shown thus far suggest that ApoC3 facilitates internalization of LNP composed of different cationic lipids (DODMA DODAP or DOTAP) into BMMC. ApoC3 activates monocytes to release cytokines^26,27^ and if mast cells are activated in the same way, they may release granules or cytokines^28^. To determine whether Apos activated BMMC, the cells were treated with ApoC3 (1,5 and 10 µg/ml) and their degranulation was measured. As shown in Fig. 3C, none of the concentrations of ApoC3 caused BMMC degranulation when compared to A23187, a calcium ionophore that activates mast cell degranulation and cytokine release by increasing intracellular calcium concentration. We also looked at TNF production and found that ApoC3 treatment didn’t induce its release as compared to A23187 stimulation (Supplementary Fig. 2). We also tested some of the other Apos including ApoB, ApoE2, ApoE4 and found that none of the Apos induce TNF release from BMMC.

### Apolipoprotein C3-mediated GFP-DODMA-LNP internalization is enhanced when BMMC were pre-treated with inhibitors of actin polymerization

**Figure 4:**
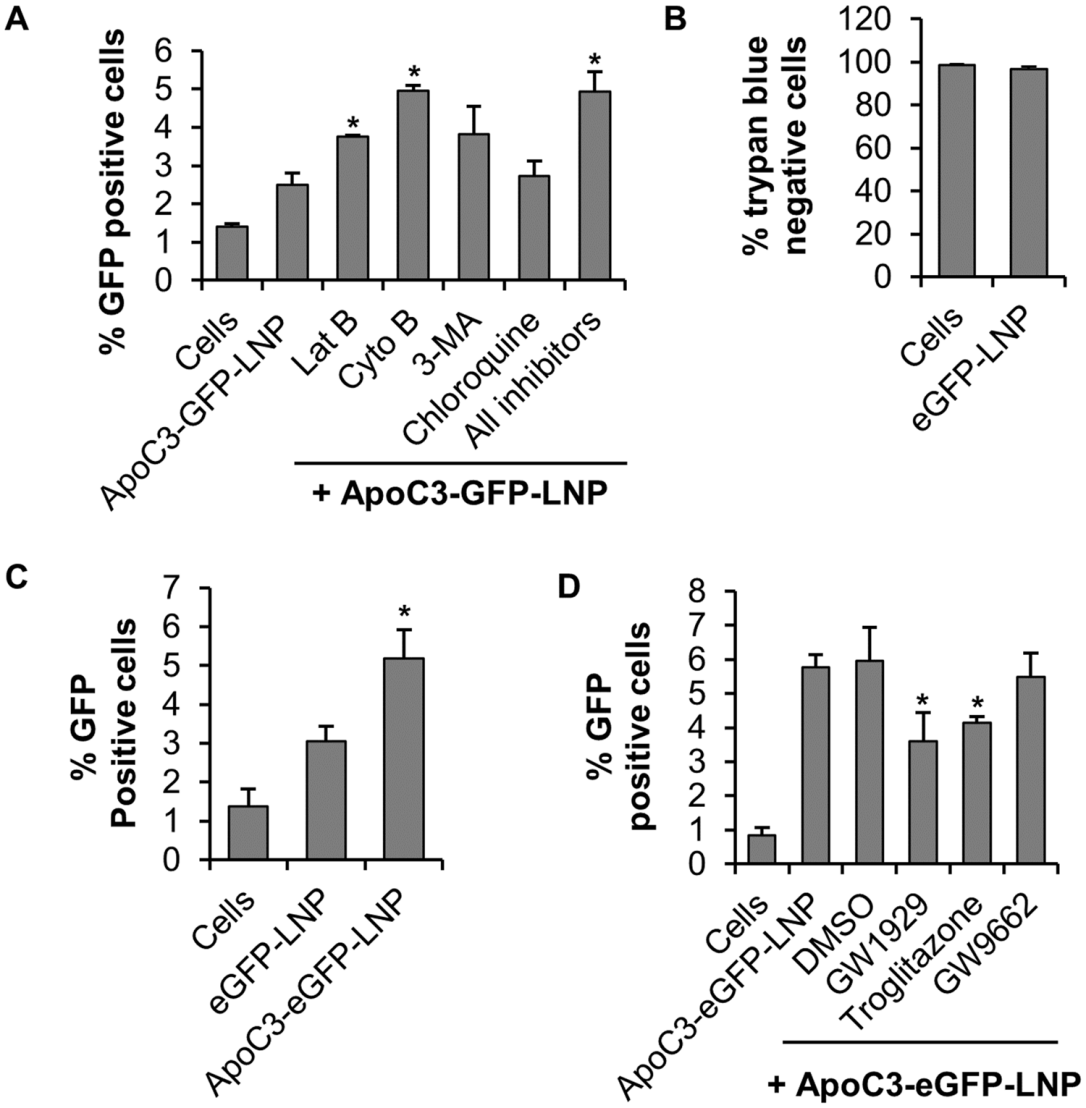
ApoC3-mediated Internalization of DODMA-LNP [encapsulating a 6.3 kb (GFP-LNP, **A**) and 4.7 kb (eGFP-LNP, **B**, **C**, **D**) plasmid expressing GFP and eGFP respectively] by BMMC is enhanced by actin polymerization inhibitors and reduced when PPARβ is activated. (**A**) BMMC were treated with 3 µM Lat B, 0.3 µM Cyto B, 600 µM 3-MA, 20 nM chloroquine or an inhibitor cocktail containing all the inhibitors for 1 h at 37 °C prior to transfection with 3 µg ApoC3-GFP-LNP for 24 h followed by flow cytometry to determine % GFP positive cells. Cells represent control BMMC that have not been treated with any endocytosis inhibitors or ApoC3-GFP-LNP. (**B**) BMMC were treated with eGFP-LNP’s for 24 h at 37 °C followed by trypan blue staining. (**C**) BMMC were transfected with eGFP-LNP or 3 µg ApoC3-eGFP-LNP followed by flow cytometry to determine % GFP positive cells. (**D**) BMMC were treated with PPARγ agonist (GW1929 and Troglitazone) or antagonist (GW9662) for 1 h at 37 °C prior to transfection with 3 µg ApoC3-eGFP-LNP followed by measuring % GFP positive cells. n = 3. *P* < 0.01 (*) is relative to ApoC3-GFP-LNP (**A**), eGFP-LNP (**C**) and ApoC3-eGFP-LNP (**D**) respectively.

The cytoskeletal network plays a prominent role in the lipid-mediated delivery of non-viral vectors to intracellular compartments^29^. To evaluate the role of the intracellular trafficking network in the internalization of GFP-DODMA-LNPs, we pre-treated the cells with various inhibitors of the endocytic pathway followed by transfection with ApoC3-GFP-DODMA-LNP. There was a significant increase in transfection efficiency as determined by % GFP positive cells when BMMC were pre-treated with latrunculin B and cytochalasin B, specific inhibitors of actin polymerization or a combination of these two inhibitors along with 3-methyadenine (phosphoionositide 3-Kinase inhibitor) and chloroquine (endolysosomal pH inhibitor; Fig. 4A). However, when BMMC were treated with inhibitor of the clathrin-mediated endocytic pathway, chloropromazine (CP), LNP internalization was significantly reduced (Supplementary Fig. 3).

### Apolipoprotein C3-mediated internalization of GFP-DODMA-LNP cargo into BMMC is independent of the payload size

Previous studies have shown that payload size does not affect the physicochemical properties of the lipoplex, but modulates gene transfer efficiency^30^. Hence, we determine whether the payload size would affect ApoC3-mediated internalization of the GFP-DODMA-LNP into BMMC. We constructed eGFP-DODMA-LNPs that encapsulated a smaller plasmid DNA (4.7 Kb instead of the 6.3 Kb plasmid DNA encapsulated in GFP-DODMA-LNP). Trypan blue exclusion assay showed that eGFP-DODMA-LNP’s were biocompatible with BMMCs with negligible loss in viability after 24 h of treatment (Fig. 4B). BMMC were treated with eGFP-DODMA-LNP or ApoC3-eGFP-DODMA-LNPs for 24 h and eGFP expression was measured by flow cytometry. ApoC3 significantly enhanced internalization of eGFP-DODMA-LNPs into BMMC (Fig. 4C) when compared to BMMC treated with eGFP-DODMA-LNP alone. A comparison of the relative increase in ApoC3 mediated transfection efficiency of GFP-DODMA-LNPs (Fig. 2B, 10.2%) to that of eGFP-DODMA-LNPs (Fig. 4C, 2.12%), showed that the former was internalized relatively better despite its larger payload size (6.3 Kb as compared to 4.7 Kb). These results collectively suggest that ApoC3 mediated potentiation of DODMA-LNP cargo delivery seems to be independent of the plasmid DNA payload size.

### PPARγ agonists inhibit apolipoprotein C3-mediated internalization of eGFP-DODMA-LNP into BMMC

Peroxisome proliferator-activated receptor gamma (PPARγ) is a nuclear transcription factor that modulates the expression of low-density lipoprotein receptor (LDLR) that is crucial in the transport and metabolism of low-density lipoprotein (LDL)^31^. Hence, we evaluated the effects of PPARγ activation on ApoC3-mediated eGFP-DODMA-LNP internalization into BMMC. Our results showed that pre-treatment of BMMC with PPARγ agonists GW1929 and troglitazone, led to a significant reduction in internalization efficiency (Fig. 4D). However, the PPARγ antagonist GW1929 had no effect on ApoC3-mediated eGFP-DODMA-LNP cargo internalization.

### PPARγ agonists reduce *Lrp8* mRNA expression and ApoER2 protein expression

**Figure. 5:**
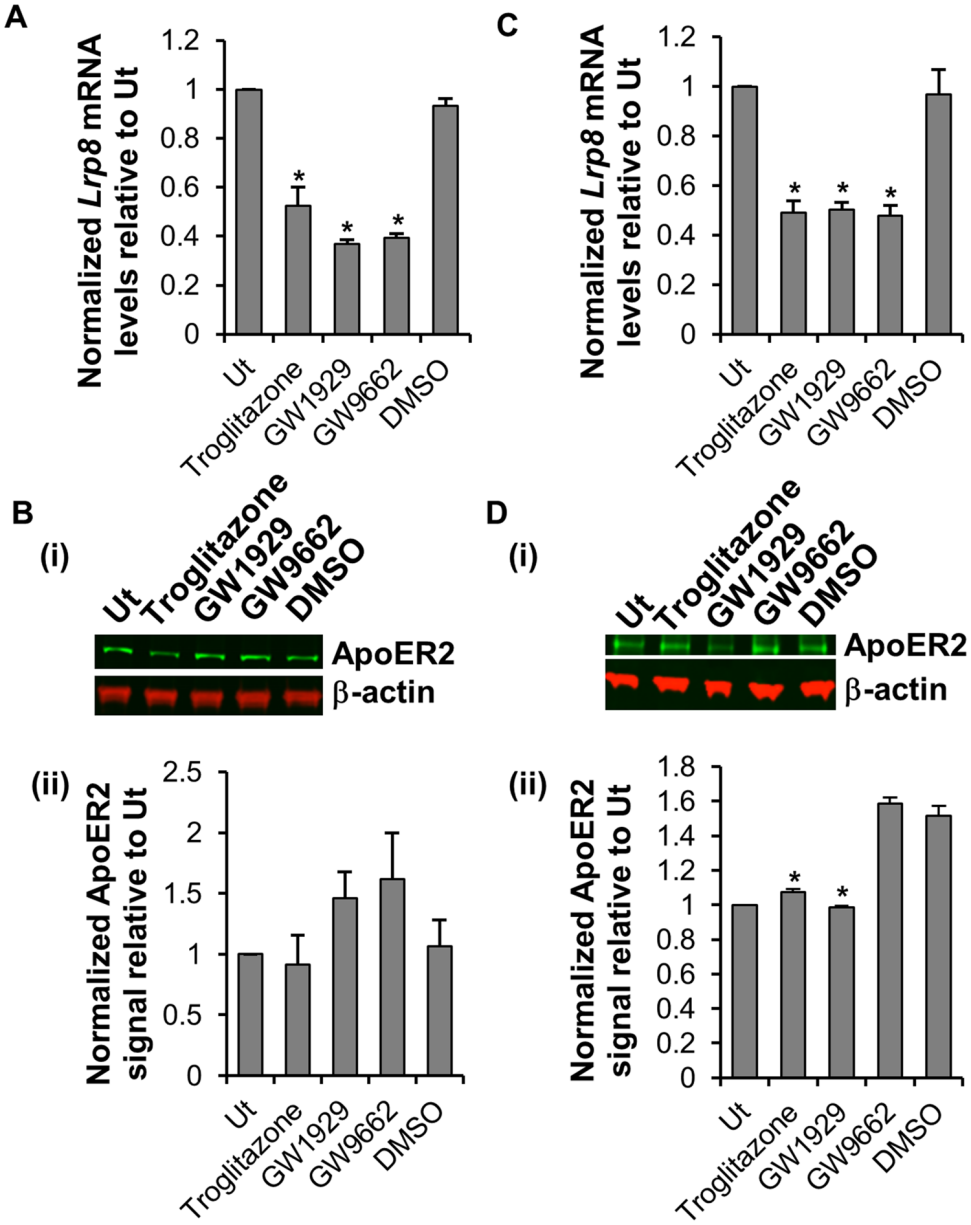
PPARγ agonists reduced *Lrp8* mRNA expression and ApoER2 protein expression. (**A**) BMMC were treated with 5 µM troglitazone, 10 µM GW1929, 5 µM GW9662 or an equal volume of DMSO for 3 h followed by RNA extraction, cDNA synthesis and qPCR to analyze *Lrp8* mRNA levels (n = 3). Glyceraldehyde-3-phosphate dehydrogenase (*Gapdh)* was utilized as an internal control to normalize the samples. Ut represents untreated BMMC. (**B**) BMMC were treated as in A for 24 h followed by protein extraction and western blot (**B-i**) and densitometric (**B-ii**) analysis using antibody against mouse ApoER2 (106 KDa). β-actin (42 KDa) was utilized as an internal control to normalize the samples. (**C**) MC/9 were treated with 5 µM troglitazone, 10 µM GW1929, 5 µM GW9662 or an equal volume of DMSO for 3 h followed by RNA extraction, cDNA synthesis and qPCR to analyze *Lrp8* mRNA levels (n = 4). The housekeeping gene *Gapdh* was utilized as an internal control to normalize the samples. Ut represents untreated MC/9. (**D**) MC/9 were treated as in C for 24 h followed by protein extraction and western blot (**D-i**) and densitometric (**D-ii**) analysis using antibody against mouse ApoER2 (106 KDa). β-actin (42 KDa) was utilized as an internal control to normalize the samples. p < 0.01 (*) is relative to DMSO (**A**, **C**, **D-ii**).

**Figure. 6:**
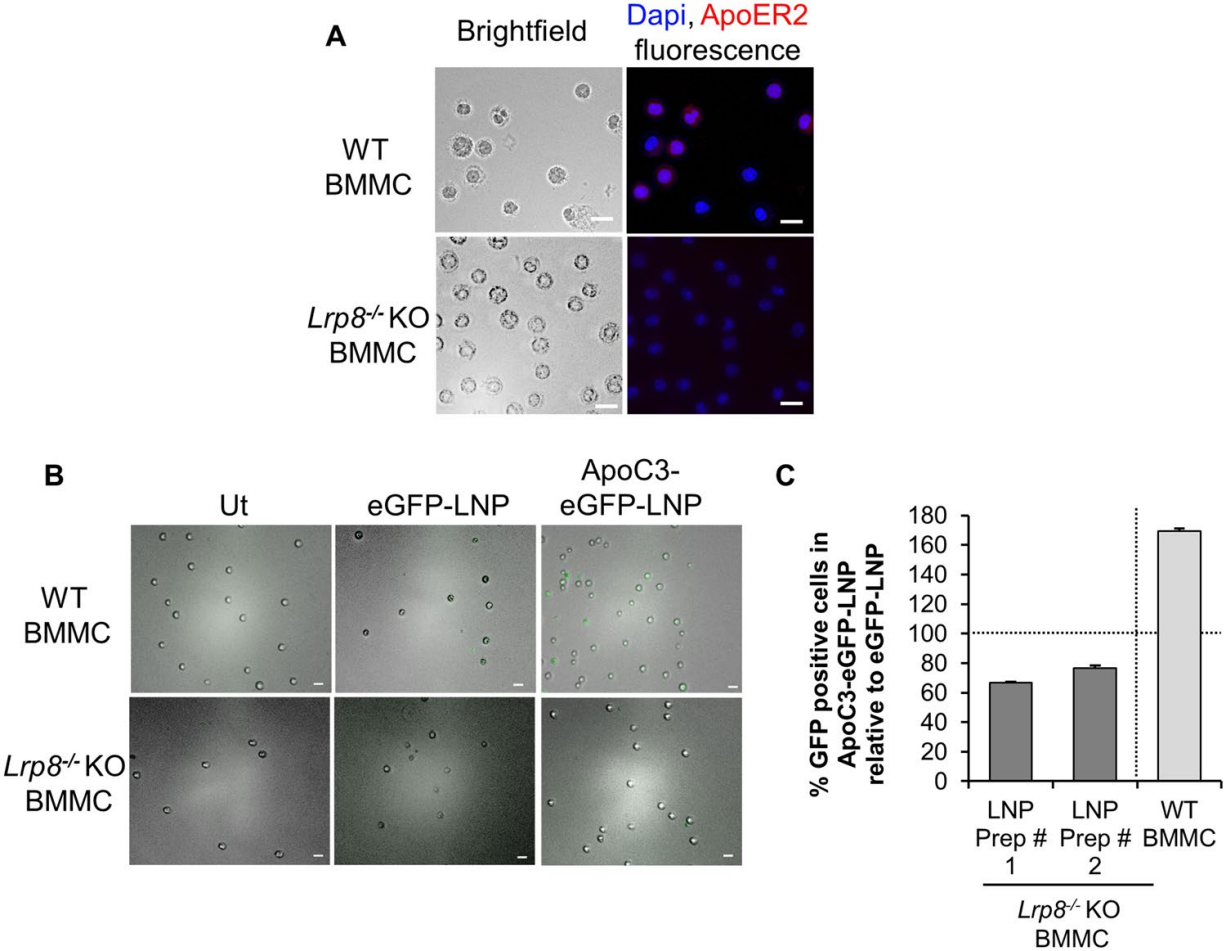
ApoC3 failed to potentiate transfection in *Lrp8*^*−/−*^ KO compared to WT BMMC. (**A**) Cell surface expression of ApoER2 (red) in WT or *Lrp8*^*−/−*^ KO BMMC. Nucleus was stained with DAPI (blue). Left-hand side panel represents brightfield images and the right-hand side panel represents fluorescent image of the same field of view. Scale bar represents 20 µm. (**B**) transfection of eGFP-DODMA-LNP (eGFP-LNP) and ApoC3 complexed eGFP-DODMA-LNP (ApoC3-eGFP-LNP) in WT and *Lrp8*^*−/−*^ KO BMMC. Scale bar represents 20 µm. (**C**) % GFP positive cells in ApoC3-eGFP-LNP relative to eGFP-LNP (100%) in *Lrp8*^*−/−*^ KO BMMC using two independent batches of eGFP-DODMA-LNP preparations (Prep # 1, n = 2 and Prep # 2, n = 2). Data from WT BMMC (n = 3) is included as a positive control.

PPARγ regulates the expression of low-density lipoprotein receptors^31–33^ such as apolipoprotein E receptor 2 (ApoER2). Although previously published microarray analysis suggested that BMMCs expressed low density lipoprotein receptor related protein 8 (*Lrp8)* mRNA^34^ that encodes ApoER2, it was unknown whether BMMC expressed the ApoER2 protein. Our western blot analysis (Fig. 5B-i, lane 1) as well as fluorescence microscopy (Fig. 6A, top panel) confirmed that WT BMMC expressed ApoER2.

The effects of the PPARγ agonists and antagonist on *Lrp8* mRNA and ApoER2 protein expression by BMMC and MC/9 was measured using quantitative polymerase chain reaction (qPCR) and western blot analysis respectively. PPARγ agonists troglitazone and GW1929 significantly reduced expression of *Lrp8* mRNA by BMMCs compared to DMSO control (Fig. 5A), but there was no significant decrease in ApoER2 protein expression (Fig. 5B-i and ii). PPARγ agonists and antagonist decreased both *Lrp8* mRNA (Fig. 5C) as well as ApoER2 protein (Fig. 5D-i and ii) expression by MC/9 when compared to DMSO.

### Apolipoprotein C3 fails to potentiate transfection of eGFP-DODMA-LNPs into *Lrp8*^*−/−*^ KO BMMC

The results shown thus far suggested that PPARγ agonists inhibited ApoC3-mediated internalization of eGFP-LNPs and that they caused a significant decrease in ApoER2 protein levels. To determine if ApoC3-mediated internalization of eGFP-LNPs was potentiated via ApoC3’s interaction with ApoER2, we performed transfection in *Lrp8*^*−/−*^ KO BMMC which do not express the *Lrp8* gene encoding ApoER2. BMMCs were cultured from bone marrows of WT (C57Bl/6) and *Lrp8*^*−/−*^ KO (C57Bl/6) mice and were found to activate optimally to release TNF using the calcium ionophore A23187 (Supplementary Fig. 4A). By fluorescence microscopy we confirmed that *Lrp8*^*−/−*^ KO BMMC did not express ApoER2 at the cell surface (Fig. 6A, compare lower with upper panel). Interestingly, *Lrp8*^*−/−*^ KO BMMC were unable to internalize eGFP-LNP in the presence of ApoC3 compared to WT BMMC as determined by fluorescence microscopy 24 h post transfection (Fig. 6B). ELISA showed that similar to WT BMMC, *Lrp8*^*−/−*^ KO BMMC did not activate to release of TNF by ApoC3 treatment (Supplementary Fig. 4B). Furthermore, for quantitative analysis of transfection efficiency, flow cytometry was conducted and % GFP positive cells in ApoC3-eGFP-LNP were measured relative to eGFP-LNP treated cells. Our results showed that ApoC3 did not potentiate internalization of eGFP-LNP from two independent preparations (Prep #1 and Prep # 2, Fig. 6C) into *Lrp8*^*−/−*^ KO BMMC.

## Discussion

Apos are regulatory proteins that bind lipids to form lipoproteins and are important in the assembly, transport and metabolism of lipoproteins in tissues by binding to membrane lipoprotein receptors^9^. Apos also maintain lipoprotein structure as well as serve as activators or inhibitors of enzymes involved in lipoprotein metabolism^25^. There are several different types of Apos that play distinct roles in lipid transport and metabolism to specific cell types throughout the human body^9^. Cationic lipid-based surface engineering is most effective in enhancing the properties of nanospheres for gene delivery^35^. The integration of Apo targeted nanoparticles as pharmaceutical carriers opens new diagnostic and therapeutic avenues in nanomedicine^36^. The intrinsic characteristic of Apos to bind lipids and facilitate their transport across different cell types in human circulation offers a very exciting tool for nanobiotechnologists.

In this study we tested seven Apos (Apo A1, B, C3, D, E2, E4 and H) for their ability to facilitate internalization of a DNA payload encapsulated in a cationic LNP into end stage terminally differentiated BMMCs. Cell cycle plays a prominent role in endocytosis and internalization primarily occurs during the proliferative stages (S + G2/M) of the cell cycle^37^. Since BMMCs are partially quiescent, only a small % of fully differentiated cells are expected to be in the proliferative stages of the cell cycle which would make them extremely difficult to manipulate genetically^23^. However, when cationic LNPs were complexed particularly with ApoC3, BMMCs were genetically modified by expressing significantly higher levels of the reporter GFP plasmid DNA (Figs. 2, 3). LNP structures were subjected to differential morphological changes when they were complexed with different Apos such as ApoC3 (~ 9 kDa), ApoB (~ 500 kDa), ApoE2 (~ 34 kDa) (Fig. 1C,D). These results suggest that different Apos bind or associate with the LNP differentially. Changes in LNP structure are associated with different biological outcomes as different Apos behave differently when complexed with LNPs (Figs. 2 and 3).

Our results show that the 6.3 Kb plasmid DNA payload encapsulated within DODMA (GFP-DODMA-LNP) and Hybrid-LNPs (GFP-Hybrid-LNP) were unable to achieve significant transfection above background fluorescence (Figs. 2B and 3B, compare % GFP positive cells of “Cells” with that of “GFP-LNP” and “GFP-Hy-LNP” respectively). However, when the size of the DNA payload was reduced to 4.7 Kb, transfection efficiency was slightly improved when compared to the background fluorescence of untreated BMMC (Fig. 4C, compare % GFP positive cells of “Cells” and “eGFP-LNP”).

When LNP preparations were incubated with different types of Apos, in particular ApoC3, transfection efficiency was significantly enhanced (Figs. 2B,H, 3B, 4C). ApoC3 promoted internalization of a 6.7 Kb plasmid DNA encapsulated in two distinct LNP preparations made up of DODMA or a combination of DODMA, DOTAP and DODAB lipids (Figs. 2B, 3B; compare % GFP positive cells of GFP-LNP or GFP-Hy-LNP with that of ApoC3 + GFP-LNP and ApoC3 + GFP-Hy-LNP respectively). Hence, ApoC3 mediated potentiation of internalization of the 6.7 Kb plasmid DNA payload seems to be independent of the composition of lipids within the LNP structure.

ApoC3 promoted internalization of GFP-DODMA-LNPs in a concentration-dependent manner (Figs. 2D,E), suggesting that binding and hence transport of the GFP-DODMA-LNP cargo into BMMC enhances with increasing concentration of ApoC3. ApoC3 also effectively increased transport of the 6.3 Kb DNA payload into a rapidly dividing mouse mast cell line MC/9 (Fig. 2H,I). MC/9 is an IL-3-dependent mouse MC line that expresses FcεRI^38^, but low levels of KIT/CD117 (Fig. 2F-ii). The MC/9 cells have often been used as model of mouse mast cells because they grow rapidly and are capable of responding to antigen activation through FcεRI by producing cytokines and chemokines such as IL-6 and TNF. Unlike BMMC, MC/9 cells do not possess mature granules that contain pre-stored mediators such as histamine and therefore, when MC/9 are activated, they do not degranulate like BMMC. By comparing ApoC3-LNP internalization by these two cell types, it is possible to distinguish the behaviour of a mast cell model with a relatively mature intracellular organelle structure (BMMC) and one with a less complex structure (MC/9). Furthermore, MC/9 divide rapidly while mature BMMC divide infrequently providing important information as to whether this approach is cell division dependent. Our data suggest that ApoC3 facilitated the internalization of DODMA-LNPs into both rapidly dividing MC/9 and quiescent BMMCs, suggesting that cells do not need to be rapidly dividing in order to be transfected with our approach.

Although ApoC3 has been shown to activate other immune cell types, possibly through toll-like receptors^26,27^, ApoC3 did not activate BMMC degranulation or TNF release, suggesting that ApoC3 may be immunologically inert to BMMC (Fig. 3D). This is an important and necessary characteristic of any LNP approach directed to mast cells.

ApoC3 is a 79 amino acid glycopeptide having a molecular weight of 8.8 KDa, that contains two amphipathic helices^39^. It is possible that ApoC3 binds or intercalates within the LNP structure via utilising its amphipathic helices. It is synthesized mostly in the liver and to a lesser extent in intestine and its expression is chiefly regulated at the transcriptional level^40^. ApoC3 plays a central role in promoting the transport of different types of lipoproteins in human body such as very low-density lipoproteins (VLDL, 30–80 nm), intermediate density lipoprotein (IDL, 25–35 nm), chylomicrons (75–1200 nm) and high-density lipoproteins (HDL, 5–12 nm)^25,41^.

Although, ApoC3 was consistently found to promote internalization of GFP-DODMA-LNP cargo into BMMC, we observed that there were differences in overall transfection efficiency from different preparations of LNP (compare transfection efficiency of ApoC3-GFP-LNP in Figs. 2B and 4A). This could be attributed to slight differences in encapsulation efficiency and zeta potential between different preparations. Regardless, it is noteworthy that even though the transfection efficiency seems to vary between independent experimental sets, the overall trend in ApoC3-mediated increase in GFP-DODMA-LNP cargo delivery is consistent.

Lipid mediated delivery of DNA payloads can be hindered by both extracellular and intracellular barriers that can have a significant impact on transfection efficiency^29^. The involvement of actin and microtubules in the intracellular dynamics of cationic liposomes have been studied extensively^42,43^. Our data show that pre-treatment of BMMC with inhibitors of actin polymerization (latrunculin B and cytochalasin B) significantly increased transfection efficiency (Fig. 4A). Cationic lipid-based nanoparticles are thought to exploit the endocytic pathway^1–3^ for internalization and the actin cytoskeleton plays a major role in intracellular trafficking and fusion of endosomes to lysosomes that form the endolysosomal compartments^44–46^, where the internalized payload is often degraded due to the acidic pH conditions. Only a few DNA molecules can escape endolysosomal degradation before entering the nucleus for transcription. Our results suggest that when actin polymerization is impaired, the LNPs are less frequently shuttled to the endolysosomal compartments and this increases their chances of escape from endolysosomes to allow for the transcription of the encapsulated DNA. Our results collectively suggest that DODMA-LNPs primarily utilize an actin-dependent endocytic pathway for internalizing into BMMC that is largely independent of phosphoionositide 3-kinase and alterations in endolysosomal pH. Nanoparticles are internalized predominantly by clathrin- or caveolae-mediated endocytosis or micropinocytosis^47^. Our results suggest that when BMMC were treated with chloropromazine, a cationic amphiphilic drug that inhibits the function of one of the key adaptor protein AP2 in clathrin-mediated endocytosis^48,49^, internalization was significantly reduced, suggesting that DODMA-LNPs may be internalized via endocytosis.

**Figure 7:**
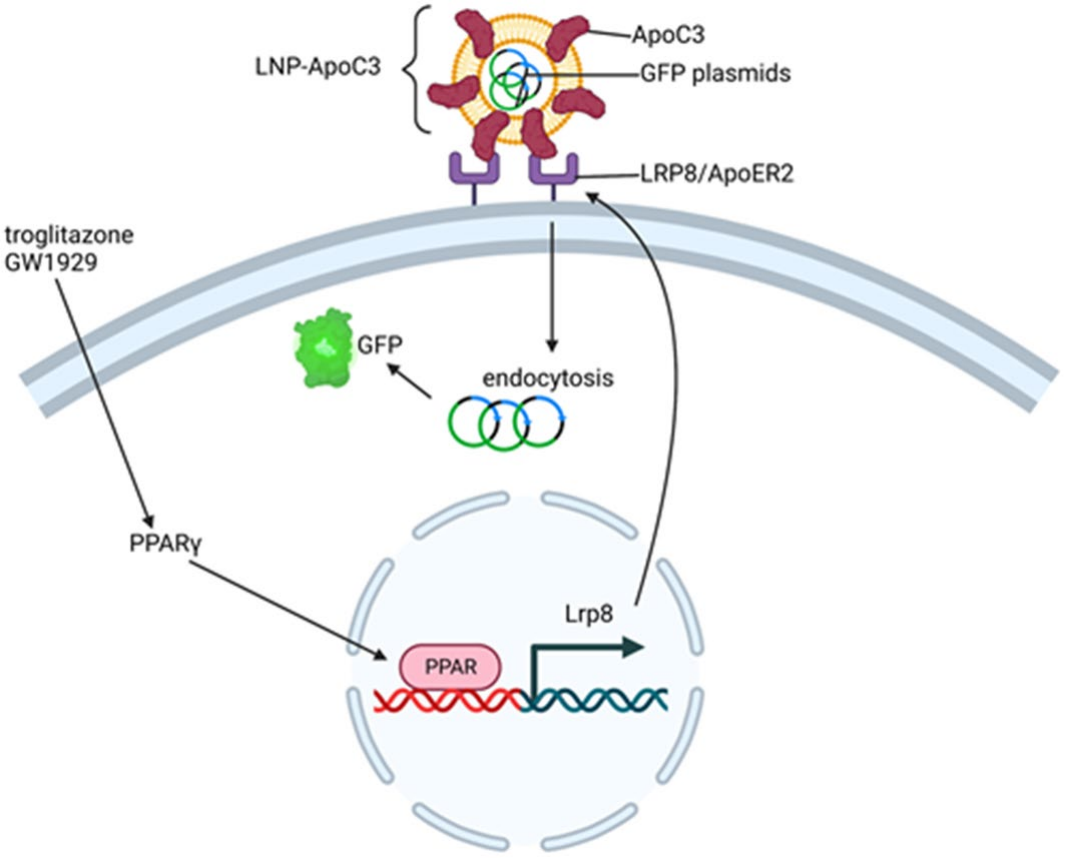
Schematic showing the role of ApoC3 in potentiating LNP internalization by interaction with ApoER2. Based on our data, we propose that one of the ways ApoC3 potentiates internalization of LNPs into BMMCs could be through interaction with the cell surface receptor ApoER2. LNPs are internalized via clathrin-mediated endocytosis that also requires actin-polymerization. Internalization results in expression of green fluorescent protein (GFP) encoded by the plasmid cargo. PPARγ is a transcription factor that can regulate the expression of many genes. Activation of PPARγ by agonists such as troglitazone and GW1929 reduces *Lrp8* transcription and causes a corresponding decrease in ApoER2 protein expression, resulting in reduced ApoC3-LNP internalization.

PPARγ is highly expressed in adipose tissue and plays a regulatory role in lipid metabolism by controlling the expression of an array of genes involved in lipid transport and metabolism in adipocytes^50–53^. PPARγ regulates the expression of low-density lipoprotein receptors (LDLR) that comprise a group of endocytic receptors (such as VLDLR, ApoER2/Lpr8, LRP1, LRP2, LRP6) on cell surface that bind and internalize lipoprotein ligands containing LDL, VLDL, IDL and chylomicrons^31–33^. ApoC3 plays a central role in promoting the transport of VLDL^25,41^. In macrophages, another type of immune cells, apolipoprotein E receptor 2 (ApoER2) also known as low-density lipoprotein receptor-related protein 8 (Lrp8), a type of LDLR, limits PPARγ expression^54^. BMMCs have been shown to express *Lrp8* mRNA^34^. Our results show that activation of PPARγ using agonists GW1929 and troglitazone, inhibits ApoC3-mediated internalization of LNP cargo into BMMC (Fig. 4D). Since PPARγ functions as a transcription regulator, we hypothesized that activation of PPARγ in BMMC may alter surface expression of lipid receptors such as ApoER2 and that could, in turn, inhibit ApoC3 mediated internalization of eGFP-DODMA-LNP cargo. PPARγ agonists troglitazone and GW1929 decreased *Lrp8* mRNA expression by BMMC (Fig. 5A) and *Lrp8* mRNA and ApoER2 protein expression by MC/9 (Fig. 5C,D). The PPARγ antagonist GW9662 decreased *Lrp8* mRNA in both BMMC (Fig. 5A) and MC/9 (Fig. 5C), although GW9662 did not have an effect on ApoER2 expression by MC/9 (Fig. 5D). It is possible that GW9662 may have non-specific effects on the PPARγ pathway that may interfere with *Lrp8* gene expression^55^. Also, since BMMC express less ApoER2 protein than MC/9 it is also possible that the effects of the PPARγ agonists are difficult to detect by this technique (Fig. 5B). It is likely that only a small subpopulation of BMMC (about 6–12%) are responsive to ApoC3-mediated LNP transfection and hence small changes in ApoER2 protein levels might be difficult to detect using western blot analysis of the entire population and even qPCR may be missing the full extent of changes in *Lrp8* expression when observed in the whole cell population (Fig. 5A). A more cell-specific approach, using either fluorescent activated cell sorting (FACS) or single cell PCR (scPCR) would be more informative and will be the focus of our future research. Moreover, ApoC3 failed to potentiate internalization of DODMA-LNPs in *Lrp8*^*−/−*^ KO BMMC. Hence, based on these data we speculate that ApoER2 may interact with ApoC3 to potentiate ApoC3-LNP internalization (Fig. 7).

Our results show that ApoC3 can play an important role in facilitating the binding and transport of cationic LNPs into BMMC and MC/9. Moreover, since ApoC3 does not activate BMMC degranulation and cytokine production, their immunologically inert nature would make them an attractive candidate for promoting transport into MC. MCs provide a convenient and effective target for anti-inflammatory therapeutic strategies due to their role in inflammatory responses, particularly in the lung and skin. Hence, based on our results, we suggest that functionalizing DODMA-LNPs with ApoC3 and co-encapsulating actin cytoskeletal inhibitors along with the payload might help achieve better transfection efficiency to genetically manipulate MCs and hence can be utilized towards development of cell directed therapeutics. Altogether, our studies reveal an important role of ApoC3 in facilitating internalization of LNPs into MCs that can have an impact on fabrication of novel nanotherapies against inflammatory diseases directed to skin or lungs.

## Supplementary Information

Supplementary Figure S1.

Supplementary Figure S2.

Supplementary Figure S3.

Supplementary Figure S4.

Supplementary Information 1.

## Data Availability

The datasets supporting the conclusions of this article are included within the article and can be provided by Dr. Marianna Kulka at marianna.kulka@nrc-cnrc.gc.ca upon reasonable request.

## Abbreviations

BMMC: Bone marrow derived mast cells
MC: Mast cell
Apo: Apolipoprotein
LNP: Lipid nanoparticle
PPARγ: Peroxisome proliferator-activated receptor gamma
ApoER2: γPolipoprotein E receptor 2
Lrp8: Low density lipoprotein receptor-related protein 8
