# Supplementary figures and images for: Apolipoprotein C3 facilitates internalization of cationic lipid nanoparticles into bone marrow-derived mouse mast cells
Source: Sci Rep. 2023 Jan 9;13:431. doi: 10.1038/s41598-022-25737-7 (PMC9828384; doi:10.1038/s41598-022-25737-7)

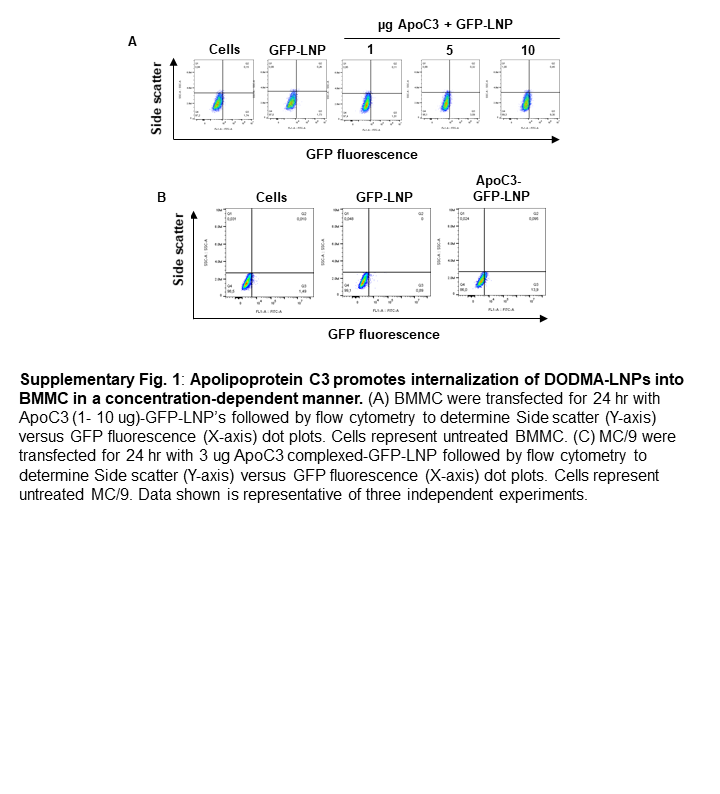

Supplement: Supplementary file 1 — Supplementary Figure S1. [file 41598_2022_25737_MOESM1_ESM.tif]

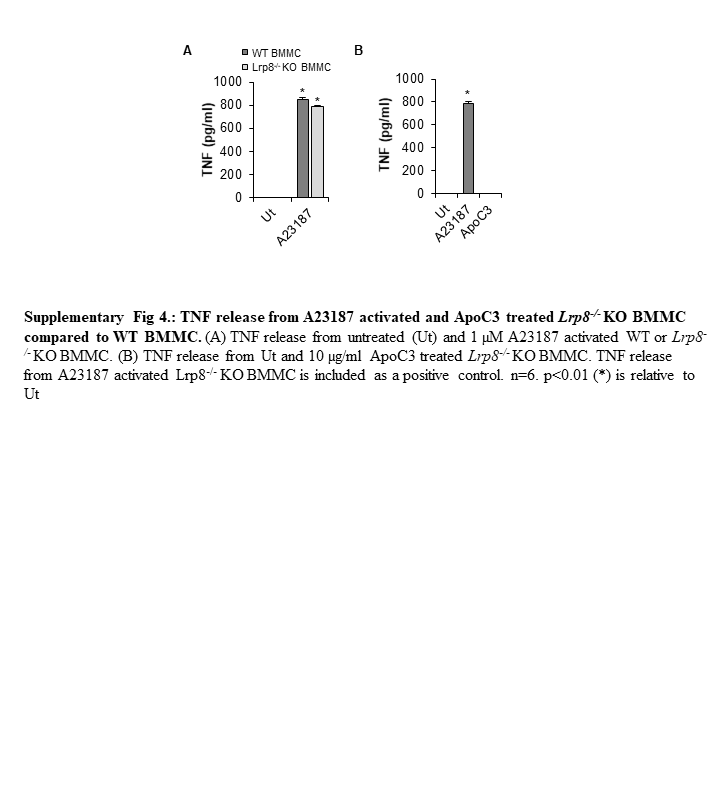

Supplement: Supplementary file 4 — Supplementary Figure S4. [file 41598_2022_25737_MOESM4_ESM.tif]
