# Supplementary material for: Apolipoprotein C3 facilitates internalization of cationic lipid nanoparticles into bone marrow-derived mouse mast cells
Source: Sci Rep. 2023 Jan 9;13:431. doi: 10.1038/s41598-022-25737-7 (PMC9828384; doi:10.1038/s41598-022-25737-7)
Supplement: Supplementary file 2 — Supplementary Figure S2. [file 41598_2022_25737_MOESM2_ESM.pdf]

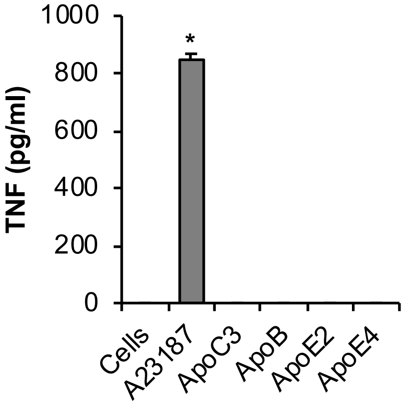

Supplementary Fig. 2: TNF release from A23187 activated and ApoC3 incubated BMMC
(A) TNF release from untreated (cells) and A23187 (1 µM) activated or 10 µg/ml ApoC3, ApoB, ApoE2 or ApoE4 treated BMMC for 24 hr. n=6. $p<0.01$ (*) is relative to cells.
