# Supplementary material for: Apolipoprotein C3 facilitates internalization of cationic lipid nanoparticles into bone marrow-derived mouse mast cells
Source: Sci Rep. 2023 Jan 9;13:431. doi: 10.1038/s41598-022-25737-7 (PMC9828384; doi:10.1038/s41598-022-25737-7)
Supplement: Supplementary file 3 — Supplementary Figure S3. [file 41598_2022_25737_MOESM3_ESM.pdf]

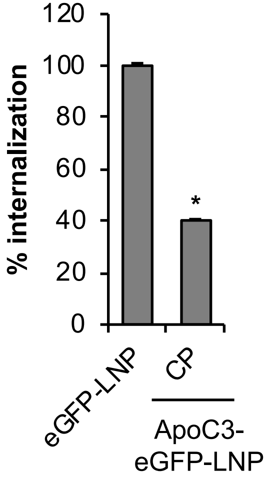

Supplementary Fig. 3: **Internalization of eGFP-DODMA-LNP (eGFP-LNP) is reduced when clathrin-mediated endocytic pathway is inhibited.** BMMC were treated with 20 μM chloropromazine (CP, Sigma-Aldrich) for 1 hr at 37°C prior to transfection with 3 μg ApoC3-eGFP-LNP for 24 hr followed by flow cytometry to determine % GFP positive cells. Data is plotted relative to eGFP-LNP. n=3. $P<0.01$ (*) is relative to eGFP-LNP.
