# Supplementary material for: Apolipoprotein C3 facilitates internalization of cationic lipid nanoparticles into bone marrow-derived mouse mast cells
Source: Sci Rep. 2023 Jan 9;13:431. doi: 10.1038/s41598-022-25737-7 (PMC9828384; doi:10.1038/s41598-022-25737-7)
Supplement: Supplementary file 5 — Supplementary Information 1. [file 41598_2022_25737_MOESM5_ESM.pdf]

BMMC cell lysate probed with ApoER2(106 kDa) and β-Actin(42 kDa)

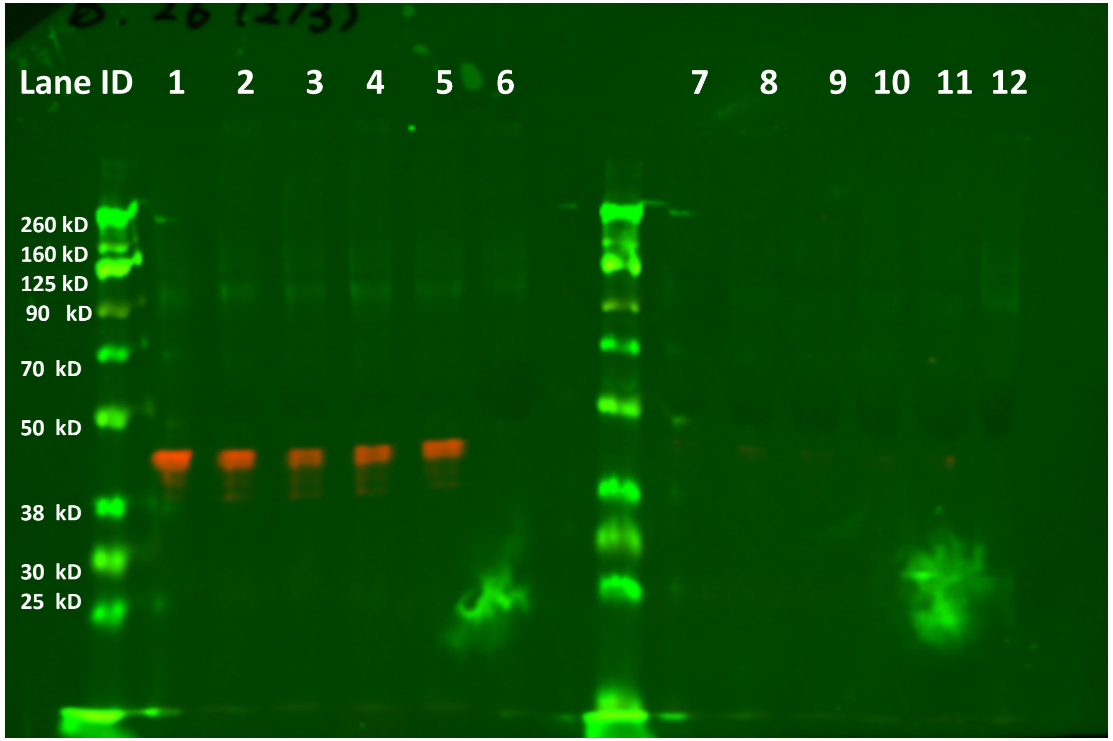

Lane ID	Sample Description
1	Untreated
2	Troglitasone (5 uM)
3	GW1929 (10 uM)
4	GW 9662 (5 uM)
5	DMSO (0.001%)

Fig. 5B-i

MC/9 cell lysate probed with ApoER2(106 kDa) and β-Actin(42 kDa)

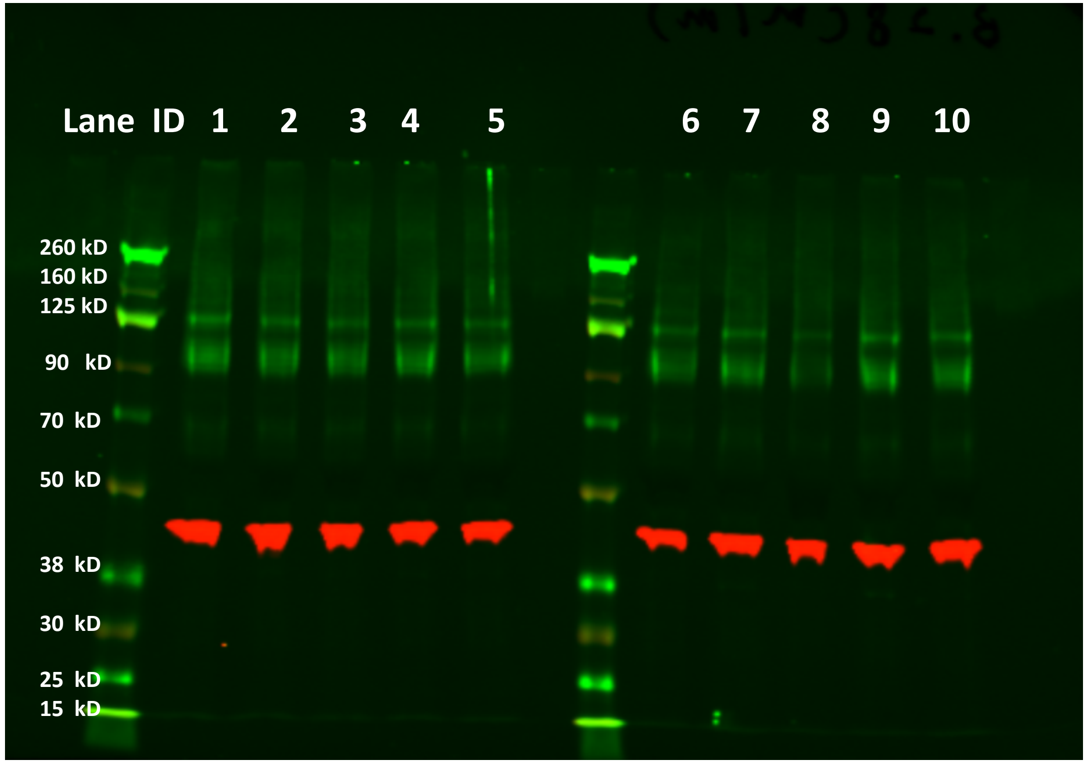

Sample ID	Sample Description
6	Untreated
7	Troglitasone (5 uM)
8	GW1929 (10 uM)
9	GW 9662 (5 uM)
10	DMSO (0.001%)

Fig. 5D-i
